# "The Hospital" Nursing Mirror

**Published:** 1898-03-19

**Authors:** 


"The Hospital" Nursing Mirror.

Being the Nursing Section of "The Hospital."

[Contributions for this Section of "The Hospital" should be addressed to the Editor, The Hospital, 28 & 29, Southampton Street, Strand, London, W.C., and should have the word "Nursing" plainly written in left-hand top corner of the envelope.]

### News from the Nursing World.

### IN MEMORIAM H.R.H. THE DUCHESS OF TECK.

On Wednesday afternoon an influential meeting assembled at the Mansion House, and passed a resolution in favour of collecting a fund for endowing a bed or beds in the Victoria Mary Ward of the Great Northern Central Hospital, in memory of the late Duchess of Teck. This ward was named after the Duchess of York at the special request of her mother, and in October last a sum of £1,050 was raised and invested to endow a bed in it, as a thank-offering for the Duchess of Teck's recovery from her serious illness. Mrs. Master, in supporting the resolution, gave an account of Her Royal Highness's pleasure when she was told of the fact. "How kind, how very kind!" she exclaimed, "and in the Jubilee year, too, when everyone has so much to do." Lady Burdett-Coutts, who has the success of the scheme very much at heart, called on the Lord Mayor, and asked his co-operation, on which he offered the use of the Mansion House for the meeting, over which he presided. Amongst those present were the Lady Mayoress, Lady Burdett-Coutts, Mr. Murdock (chairman of the Great Northern Hospital), Mr. Burdett-Coutts (treasurer), and Mrs. Harkness (hon. secretary of the Ladies' Association).

### NEW NURSES' HOMES, LIVERPOOL AND BRADFORD.

Liverpool has just spent £11,350 on a new home for the nurses employed at the workhouse. In the basement are the dining and cloak rooms, with kitchens, servants' hall, and pantries. On the ground floor are two large sitting-rooms, divided by Stokes's patent folding-doors, so that they may be converted into a large lecture hall or meeting-room at will. There is also the matron's-room and 18 bed-rooms. The two storeys above are devoted to sleeping apartments. There is bath and lavatory accommodation on every floor, and the closets are in a sanitary tower separated from the main building by ventilated passages. A wide corridor runs the length of the home, and has stone staircases at either end. The whole house is lighted by electricity and heated by hot water. The new home at Bradford Union cost £7,000. There are rooms for 36 nurses. It is built on the pavilion plan, and provides means of exit in case of fire from the workhouse.

### NURSE VICTIMS OF THE PLAGUE.

Further particulars have been received respecting the deaths of Miss Morgan and Miss MacDougall of the plague in India. Miss Morgan, it appears, was at Poonah taking charge of the hospital in the temporary absence of Miss Wheatley in Bombay, when she became ill. At first she suffered apparently from a slight chill after a bicycle ride, but graver symptoms soon set in, and in spite of every attention she succumbed to the disease. She was buried with all possible honours. A large proportion of those engaged in plague duty were present, and amongst the many beautiful wreaths was a cross of bright red geraniums, which was placed on the coffin. We shall look forward to hearing fuller details of the disastrous fire that broke out in the Parsee plague hospital at Poona. The nurses' loss, which was considerable, has evoked great sympathy, and subscriptions to the fund for replacing it are coming in freely.

### THE NORTH LONDON NURSING ASSOCIATION.

There was a large attendance at the annual meeting of the North London Nursing Association, at which the Rev. W. Tyndale Hollins took the chair. One thousand three hundred and one cases were nursed, an average of 108 per month, of which 500 were recommended by medical men. As soon as funds permit the staff, of which the number varies from six to eight, will be increased to ten, for which number there is accommodation in the home. Unfortunately, considerable anxiety is caused by the balance again being on the deficit side of the ledger, and special measures will be taken to invoke the aid of added subscriptions.

### FEMALE MEDICAL AID FOR INDIA.

The Marchioness of Dufferin and Ava sends us a copy of the annual report of the fund which bears her name. The actual receipts of the central fund are small, and this prevents the committee extending the benefits of the work as they could wish. There is nothing, however, to regret in the way the movement has been taken up in India. Native rulers have given it a cordial welcome, and it is only natural that local authorities should give their subscriptions to their own hospitals. Nevertheless, it is the work of the central association that makes the work national. It grants scholarships for the training of promising pupils; it also comes to the aid of hospitals in need of a helping hand. There are now at work in India 28 ladies of the first grade, that is, those whose names are on the English medical register, 70 assistant surgeons, and 70 hospital assistants; and 1,327,000 native women have received medical aid from women during the year just closed. The income is only £4,000 a year from invested funds, and it is hoped that subscriptions will be sent to the United Kingdom branch, which strives to advance every effort to extend medical work through the vast continent of India.

### THE EAST LONDON NURSING SOCIETY.

The East London Nursing Society comes before its supporters with an excellent record. It is free from debt, and has money in hand to relieve the executive committee from anxiety for the coming twelve months. Its tale of work is large—5,089 cases. The annual meeting at the Mansion House on Friday, 11th, was presided over by the Lord Mayor, and several important and influential supporters were present.

### THE WORKHOUSE INFIRMARY NURSING ASSOCIATION.

We have before us the report of the Workhouse Infirmary Nursing Association for 1897. It shows plainly that the need of the work it so admirably performed is greater than ever, and that Guardians have the utmost difficulty in obtaining reliable nurses. Out of 113 applications received for nurses only 43 appointments could be filled. The association has removed from 6, Adam Street, Strand, to 49, Victoria Street, Westminster. Miss Wilson, for many years the energetic hon. secretary and treasurer, has resigned the arduous work connected with the former post, but still continues to be treasurer. Miss Gill undertakes the duties of secretary. Five of the nine probationers now in training are to receive a three years' course which will qualify them for the position of superintendent nurse.

### DR. PLAYFAIR AND ANTISEPTICS.

Dr. Playfair does not approve of monthly nurses being left to choose their own antiseptics, as he is of opinion that no nurse would willingly use carbolic solution of sufficient strength to be of any good, as it would roughen the hands, and that, if left to their own devices, they might prefer a few drops of Condy's fluid or some such innocent mixture from a microbe's point of view. He recommends the 1 in 1,000 perchloride of mercury solution, which anyone can carry about in the form of a tabloid, and which is bland and non-irritatng to the skin.

### BIRMINGHAM DISTRICT NURSING SOCIETY.

Birmingham has the name for being the best municipally governed city in England, and the comprehensive scheme devised by the District Nursing Society is worthy of such a reputation. It is, in brief, that the whole city should be nursed by one society, established in four homes in the north, south, east, and west, accommodating from eight to ten nurses in each. This would give one nurse to every 10,000 inhabitants. In spite of difficulties, and they are not a few, there is every hope that success will crown this laudable ambition. The want of money, of course, is the chief obstacle; but when it is taken into consideration that the present funds are subscribed by about 150 persons only, and that the work of the nurses commands a growing sympathy and appreciation, it ought not to be an insurmountable one. The Diamond Jubilee grant of £2,500 has given the expansion of the work an impetus, and it has been expended in the purchase of a freehold house near Highgate Park, Moseley-road. The annual meeting was well attended, and the report showed that there were 15 nurses now at work, and a balance in hand of £94. The cost of each visit is slightly under sixpence, and the average cost per case is 12s. 6d.

### NURSING IN SURREY VILLAGES.

Five parishes unite to form what is known as the Shottermill and District Nursing Association, and the record of the year's work has been forwarded to us by the sister-in-charge. The system is that of the Holt-Ockley. The nurses, as a rule, have a year's training in a general hospital, and two months in a lying-in hospital. They live in a central home and are sent out to cases in the affiliated parishes at a uniform charge of 15s. a week. When at liberty they undertake other than cottage patients. Some of the nurses of the staff are fully trained, but there is difficulty in finding women who are fully trained, and are also willing to undertake the ordinary work of the cottage home to which they are sent. About £100 a-year is needed from private subscriptions to meet all expenses.

### A CAUTION TO NURSES.

An apparently innocent custom, which has grown to a large extent in some foreign countries, but in favour of which nothing can be said, is finding its way into England. It is nothing more nor less than selling on commission. So far, if one may judge by the advertisements in the papers, it is confined as yet to light-pursed gentry who eke out a livelihood by trading on their friends in a matter of wine and cigars. That is bad enough, but when the danger threatens to invade the nurses' calling, and when any trained nurse who reaches the patient's bedside may come as an agent for a special firm of invalid appliances, it is time to raise a protest. There is nothing dishonourable in trade, but no one wants to be nursed by a trading agent who, in addition to her pay, pockets a handsome percentage on all the dressings and luxuries necessary in the sick room. The abuses that would of a certainty creep in are too numerous to mention. Suppose, for instance, that the doctor orders Brand's essence and the nurse is an agent for some other of the numerous and excellent meat essences on the market, is she going to give up what she, perhaps, considers her just perquisite for what she may regard as the doctor's whim? And will doctors put up with such a system? A nurse who is known to engage in this kind of thing will sooner or later find herself tabooed alike by medical men and patients, and will find, like the dog in the fable, that she has lost the substance for the sake of a shadow.

### NURSING IN STAFFORDSHIRE.

Jubilee year has been one of great prosperity for the Staffordshire Institution for Nurses. Work has been plentiful, and the health of the nurses excellent. Only four cases of slight illness have occurred amongst the large staff of 124, which includes district nurses and probationers. In spite of having nursed 16 cases gratuitously and 12 at reduced terms, the earnings reached the large sum of £4,787. According to a resolution passed at the last annual meeting, the nurses received 10 per cent. of their earnings as a bonus. In the steady growth of the institution the committee acknowledge the influence of the able lady superintendent, Miss Shirley, who has held that position for 14 years, and who continues to hold it to their great content.

### SHORT ITEMS.

In American training schools each nurse upon obtaining her certificate is presented with a set of surgical instruments.—The love of flowers seems inherent to nurses. In the wilds of Buluwayo the universal meat tins and jam jars are made to do duty as flower-pots, and are filled with beautiful and luxuriant native plants. Those used for indoor decoration are covered with crinkled paper and other ornamental devices, but those outside retain their original lables relating to marmalade and pickles producing a decidedly curious effect from a botanical point of view.—The Belfast Society for Providing Nurses for the Sick Poor has certainly achieved a most successful financial record. It begins the new year with £1,082 in hand to meet the expenses of the coming year. The majority of deaths were in patients suffering from consumption and cancer. Much help has been given by lending nursing appliances, and sometimes beds to the invalids.—An extraordinary rumour comes from Birmingham—namely, that nurses are not allowed to cycle. The prohibition is the more incomprehensible as the nurses have not adopted any "advanced" garb, "bloomers" being unknown. Such an unpopular and short-sighted decree can be only short-lived.

### Antiseptics for Nurses.

By a Medical Woman.

### V.—COMPOSITION AND USES OF SOME OF THE ANTISEPTICS IN COMMON USE.

### Compounds of Mercury.

Another antiseptic very commonly used in surgery is perchloride of mercury, or corrosive sublimate. For a year or two after its introduction by Sir Joseph Lister in 1889, it was a formidable rival to carbolic, and is, in fact, the only antiseptic that can at present at all compare with carbolic in efficiency. It has, however, several serious disadvantages; one of these is its extremely poisonous properties. On the other hand, it is such a powerful antiseptic that it can be used in weaker solutions, and is not so bulky for the district nurse, or for operations in private houses. It is found that a solution of 1 part corrosive sublimate in 5,000 parts water will destroy all living organisms in water, and this strength can be used for washing recent wounds, whilst a solution of 1 in 2,000 or 1 in 1,000 can be used for irrigating wounds during an operation, for cleansing hands, &c. It cannot, however, entirely replace carbolic acid, since it cannot be used for instruments, as it acts chemically on the metal. Erichsen considers the 1 in 1,000 solution of corrosive sublimate to be equivalent to 1 in 20 carbolic acid, and 1 in 2,000 sublimate equal to 1 in 40 carbolic; but in prolonged operations, or for irrigating a large wound, it is better to use more dilute solutions. On the other hand, for foul abscesses, or for cleansing the unbroken skin, an even stronger solution may be used. It is important to remember that corrosive sublimate will not act if the skin be greasy, and consequently the skin must first be thoroughly cleased with plenty of soap and hot water, and then dipped in a weak solution of ammonia, or even in methylated spirit, to remove any possible trace of greasiness that may remain.

If solutions of corrosive sublimate are made with ordinary water, after a while there is an insoluble deposit of lime salts formed, and if the water is very hard, this deposit may amount to as much as 50 per cent. of the perchloride present. Hence all solutions that are to be kept for any time should be prepared with distilled water if possible. It has been found that if an equal quantity of salt be added this precipitation is prevented, and the character of the solution remains unaltered. Perchloride is now made in patent tabloids or solids, mixed with a proper quantity of salt; these dissolve readily in water without forming a precipitate, and the tabloid in a pint of water makes a solution of 1 in 1,000 strength. Perchloride of mercury must be cautiously used as it is specially poisonous and irritating. In a 1 in 1,000 solution the maximum medical dose is contained in about four and a half drachms. Hence in wounds or cavities that have to be frequently syringed out with a solution of this strength great care must be taken not to leave behind a poisonous dose each time. It must also be remembered that some patients are extremely susceptible to mercury, and cases of poisoning by corrosive sublimate are not very uncommon unfortunately. The symptoms are those that one might expect from the action of such a powerful irritant, viz., great pain in the abdomen, with diarrhœa and bloody motions, death generally resulting from the exhaustion and collapse caused by the diarrhœa. There may also be inflammation and some superficial ulceration of the small intestines. According to Erichsen there is, curiously enough, very rarely any salivation. Very occasionally there is some local irritation from the dressings characterised by redness, some vesication, or even a superficial inflammation like eczema. From a surgical point of view, it is a great drawback that perchloride forms an insoluble substance with albumen, but this redissolves if excess of perchloride is used.

The dressings prepared for this antiseptic are the following: Wool, prepared by soaking absorbent wool in a solution of 1 part perchloride, 50 parts of glycerine, which increases the absorbing power of the wool, and 450 parts of water, and dried. Many absorbent substances, such as jute, peat, sphagnum moss, tow, sawdust, &c., have been similarly prepared.

Another preparation is wood wool, which is prepared by tearing and grinding pine wood by machinery, and then soaking it in perchloride and drying. It makes an excellent application in the form of pads covered with sublimate gauze. This preparation has the great advantage of softness, elasticity, and great absorbent powers. In Germany, sphagnum moss is soaked in perchloride, dried, and used as we do wood wool.

Sublimate gauze is made of the strength of 1 in 200 or 300. Perchloride "papers" have been invented, which consist of sheets of unglazed paper, perforated like postage stamps. The papers are prepared by dropping on to them a solution of perchloride of standard strength, after which the papers are dried and made up into books. When required, the paper has only to be moistened and applied to the wound.

One patent disinfectant, known as "St. Bede's Disinfectant," is made with perchloride of mercury, ordinary sulphate, a little sulphuric acid, eucalyptus, and thymol. It is coloured with indigo, and made up into little blocks.

Sal alembroth is another form of perchloride of mercury, in the double chloride and ammonium. It is said to be less irritating and not to form insoluble compounds with blood serum. It is, however, so soluble that it is readily washed out of the dressings if there is much discharge from a wound; but for small wounds, or where there is not much discharge, it acts very well. The blue colour of the gauze and wool is merely due to aniline blue, by which they are distinguished. Before application the gauze is wrung out in a solution of 1 in 2,000 perchloride, and over this is applied several layers of dry gauze.

Cyanide of mercury and zinc was introduced by Lister in 1887, and is still very generally used. Though insoluble in water, this salt is soluble in blood serum, and does not form an insoluble compound with albumen; hence it is very valuable as an antiseptic. Moreover, it very rarely indeed produces any irritation around the wound.

It is used in the form of gauze, which must be soaked in and wrung out of carbolic acid solution—1 in 20—and not in perchloride, as this would change it into an insoluble salt and would destroy its most valuable property. Over the wet gauze must be placed several layers of dry. This gauze is extremely absorbent and soft, and is a very satisfactory dressing. It is usually coloured mauve, for the sake of distinction, with an aniline dye called rosolane, which also fixes the double salt in the gauze.

The only other preparation of the double cyanide is a "cream," which is made by simply mixing sufficient carbolic solution 1 in 20 with the dry salt to make it into a creamy consistence.

Another form which is employed by some surgeons, but is not in such general use as the preceding, is biniodide of mercury, and it is claimed that it has none of the disadvantages of the preceding. It does not coagulate albumen, and, if left behind in cavities, it is said to produce no poisonous symptoms, and it is claimed for it that its penetrating powers are greater than those of either carbolic or corrosive sublimate. It also has been prepared in tabloids of such strength that two dissolved in one pint of water gives a solution of 1 in 4,000 strength. Unlike corrosive sublimate, it does not form a precipitate in ordinary tap water.

### The Training School for Nurses.

Read at the Opening of Lakeside Hospital, Cleveland, Ohio, January 12th, 1898, by Mrs. ROBB (Miss Isabella Hampton).

IT was in a military hospital, amid the tumult of battle in the great Crimean war, that the modern system of nursing found its birthplace. Here came into existence a force born to do battle for evermore in the never-ceasing struggle for life. As the child of that war has increased in years, it has grown mightily in strength, until to day, although still young, its power is recognised in the east and west, the north and south. There is hardly a mission in foreign lands that has not its skilled nurses. In plague-stricken India, in the wars of both East and West; in the South with its fatal fever, and in the lands of the North, wheresoever plague, pestilence, or famine are to be found, there, too, side by side, is the trained nurse, quietly, silently doing battle. Even in our everyday lives we can scarcely turn our eyes in any direction without encountering her. There is not a modern hospital of which she is not an integral part. Scientific medicine would find it hard to do without her. In infirmaries, asylums, day nurseries, schools, church parishes, in the solitary sick-room, and among the poor in their homes she is doing her work. To few is it given as to Florence Nightingale, the heroine of the Crimea and the Patron Saint of Nurses, to see so great results in so brief a time from the seed she has sown. And it is the light of her lamp at night that still throws its shadow on the wall of many a hospital ward the world over, for none among her followers have a clearer or more penetrating insight into things pertaining to hospitals and nursing than has Miss Nightingale to-day. Long may that light with its inspiring flame burn among us.

Something over eight years ago, in connection with the opening of that hospital which is the heart and centre of scientific medicine in America, and in the possession of which medical men, the continent over, feel a truly pardonable pride, it was my privilege to speak briefly of the aims and objects of training schools for nurses. These eight years have seen many changes in the thought, work, and methods of the medical profession—changes which have necessarily exercised a great influence upon the training of nurses.

At the present time it would be considered out of date and only a sign of ignorance for a physician to affirm that all he requires of a nurse is that she should be a well-working automaton to carry out his orders, and that he would prefer that she should not know even part of his reasons for what is done for the patient. Such an idea, popular as it once was, has now, fortunately, become antiquated. The progress of medicine itself has taught that a very different intelligence is required of a nurse. The science of bacteriology, which in a few years has conclusively demonstrated the causes of many diseases, and her sister, "preventive medicine," require one and all who meddle with medicine, whether as physicians or nurses, to be people of intelligence. So long as drugs were looked upon as "cure-alls" for all bodily ailments, an automaton who would administer the doses at certain intervals may have perhaps answered every purpose, but at present some of our remedial or preventive measures would seem to the uninitiated to appear too vague and even far-fetched, so that it would be impossible to insure a conscientious carrying out of them unless the intelligence were appealed to. What, for instance, are we to understand by cleanliness? For the uninitiated the sense is very clear. And yet there is a deeper meaning which can only be appreciated by those who have mastered at least the broad principles of bacteriology. How hopeless and dull, not to say irritating, would be the many washings and the various aseptic precautions which are now required from the nurse by the physician unless she had learned from bacteriology to appreciate the fact that there exists a surgical, a microscopical cleanliness. For this purpose then the nurse must have some knowledge of the broad principles of bacteriology. To fully appreciate the effect upon the patient of the air he breathes and the food he eats she must know something of ventilation and hygiene. For the proper care of the body she has to go to physiology. To become acquainted with the best forms and preparations of food by which the greatest possible amount of bodily resistance to disease are established and maintained she must profit by the demonstrations given in the diet school. Such matters of detail are usually entrusted to the nurse. She alone can devote to them such constant and unremitting attention as are necessary. It is true that the physician can lay down a broad general outline of such matters, but the details—the little things that matter so much—must of necessity be often left to the nurse, and how shall she supply all this without intelligence and a proper education in her profession? In hospitals the doctors may be temporarily off duty, the nurse is always in charge. Her candle goeth not out by night, and it is to her watchfulness and intelligent care that the patients are in the main committed through the night. She must be taught how much and how little she can do, and when it is necessary to call in further aid.

But the training of a nurse in a hospital does not deal merely with book-learning or with clinical methods. It carries with it the building up of character. The nurse is taught that the most scrupulous honesty is necessary in all her work, that any omission, whether it come about wilfully or through ignorance, may be productive of the most serious results; that one step in aseptic technique omitted or slurred over may be paid for by the life of the patient committed to her charge. May we not then regard the training school of a hospital as a place not only for preparing women to undertake properly the care of the sick, but also where properly selected women are given such moral and educational advantages that they may go forth equipped to aid in the practical solution of some of the various social problems which can be solved only by the help of intelligent womanly work. She is limited only by her limitations, for as she shows her ability to fill them, other fields of usefulness are being opened up to her.

A further progress or evolution from hospital, private nursing, and district nursing dates from four years ago, when two trained nurses in New York City formed a nurses' settlement in one of the most densely populated tenement districts. From rooms in a tenement house the headquarters have been removed to a good-sized private dwelling in which eight nurses now make their home. At present there are three such settlements in New York which do an infinite variety of work besides attending upon the sick. The members of these settlements have become a power for good among much misery. Their opportunities for the acquirement of practical knowledge concerning the problems connected with the poor in all great cities has lately been recognised by the election of one of their number as a member of the Sanitary Health Association of New York City, while in London a trained nurse has just been appointed on the London School Board. In the same city a nurse is regularly employed by the Corporation to teach practical hygiene in the poor districts.

Another movement of interest is that which aims at the introduction of trained nurses in families of only moderate means. In some cities associations have been formed to provide a nurse who attends for one or more hours a day for a moderate fee, and who sees that such arrangements are made that the patient is taken proper care of.

Thus it will be seen that in many ways the profession is broadening out, and the graduate members are practically taking their part in aiding in the propagation and advancement of preventive medicine.

Within the profession itself also we are not standing still, but are ever aiming at improvements upon older methods. We have now an organised association of superintendents of training schools, who, at the expenditure of not a little time, energy, and money, are doing much to bring about some degree of uniformity among the different schools. Their aim is to establish, as far as possible, uniform standards as regards requirements for entrance, the curriculum, and the granting of certificates. The best schools are beginning to increase the time of training from two to three years, and are thus improving the quality of the graduate nurse. If they have lessened the quantity by these means they have done no harm. This distinct effort to put training schools upon a higher educational basis has also been furthered by those schools who have at the same time introduced the non-payment system, and who, while asking the nurse to forego a present remuneration, have guaranteed to her in return for her work a thorough education as well as practical training. Under these circumstances it was only just that more time for study, lectures, and demonstrations should be given to the pupil nurse, and that all her energies should not be exhausted in the routine work of the wards, which should now occupy her only for eight hours out of the twenty-four. While some schools have extended their course to three years, and others have adopted the non-payment system, it is to be regretted that at present in only one or two hospitals have the hours of daily practical work been reduced to eight, the average time required still being usually from ten to eleven hours.

As a nurse who has watched all these changes with keen interest, I take peculiar pleasure in assisting at the opening of the Lakeside Hospital, because it is the first hospital on record supported by private contributions that opens its school for nurses upon a purely educational basis. The women are to be congratulated who enter this training school as pupil nurses. The fact that the trustees of the hospital in its construction and sanitary arrangements have given every thought for the care and well-being of the patients augurs well for the fulfilment of the obligations which they have taken upon themselves in accepting pupils in their training school. Honour and credit are due to their broad-minded and far-seeing policy which insists that patients can be properly taken care of only by intelligent and well-trained nurses. This school offers to its pupils a three years' course of instruction with eight hours practical work in the wards. The pupils receive no payment, but are furnished with a thorough, systematic, practical, and theoretical education in return for their work. As a result the women who enter as pupils will be those who come seeking knowledge and who have high ideals. The commercially-minded woman who looks only to the dollars and cents. can have no place here. Fortunate, indeed, are those whose privilege it is to study nursing at this time and to become a part of the working force of such a hospital. Of friends she finds plenty, of enemies few. She cannot well be classed with the "new woman," for her art is ancient history and extends through the ages. We read of fair ladyes nursing their stricken knights, of Elaine tending the wounded Launcellot, or of the Good Samaritan binding up wounds, pouring in oil and wine. Nor do we lack the Master's reply to the question, "When saw we Thee sick or in prison and ministered unto thee?" "Even as ye did it unto the least of one of these my brethren." The woman in this profession needs no pity bestowed upon her; her days are all rounded out in absorbing work and interest, and so are happy ones. It is exceedingly interesting to visit old St. Bartholomew's Hospital in London. Over 500 years have passed since its doors were first opened to the sick. Its history is most impressive, and as one follows it step by step through its buildings one realises, as never before, that such a hospital is not only for to-day and to-morrow or for our own short lives, but that its life and influence will extend far into the future and will bear with it the lives of its men and women bound up with it.

To the building up a fabric of personal education and personal character; to the preparation for boundless opportunities for good work in the world, to happy, useful lives, and to the welfare of future generations are the women dedicated who become part of this new hospital and training school, the inception of which we are here to witness. As an older member in this profession of nursing I rejoice in its high standing and the opportunities offered to its nurses, and as the representative of the associated trained nurses in the United States and Canada I bid it welcome and God-speed.

### The Diary of a New Pro.

(FOUNDED ON FANCY.)

THE following little skit, which appeared in the February number of the *Guyoscope*, is so amusing that we have reprinted it from the columns of that clever little magazine, which is now appearing in a new form:—

MONDAY, JANUARY 31ST.—I have settled on arriving in my new sphere of usefulness at Guy's to keep a *diary*, in which I shall regularly note down *interesting events*, and any *thoughts* suggested by my work. Really my first day here has been *full* of incident; in the first place, a girl in a blue dress, who seems to be the head of the ward, told me to polish some *horrid* brass things which made my hands in an *awful* mess. What this has got to do with nursing I fail to see! I had quite an adventure because I was sent to fetch some milk, and I got into an underground passage there is (a *horrid* place), and at last I found some little spiral stairs, which were *awfully narrow*; and when I got to the top I saw a kind of archway leading into a courtyard, and there were several young men standing there who *stared* at me horribly; so I asked one of them whether this was the way to the kitchen, but he smiled and said it was the college, and that he would take me back as he happened to be going that way himself! He took me all the way back, and was really *quite* a nice boy, though he looked *very* young. I think he must have been what is called a "house doctor," for he seemed to know his way about so well! But *imagine* how *awful* I felt because when I got back I found I could not get into the ward, and there was a notice put up that it was closed till half-past one. I *waited* and *waited*, and it was *so* trying, and at last I had to sit down on the stairs and cry. And then someone came up and somehow they explained that I was a new pro., and got the ward opened for me, which was very nice of them. I had a lot of other nasty things to do which weren't nursing or anything *like* what I expected it was going to be, a bit; and the stupid sister made me late for my dinner, though I *told* her I had to go. I *do* think she must be a *very* unbusiness like person. Oh! I nearly forgot to put down that when I was trying to find the nurses' dormitory I got into a place I felt *sure* was the right one, but it wasn't really after all. Of course I stuck to my point, till a tall sister came and told me she was sure I was wrong, because it was her ward, so then I had to go. She gave me a back number of the *Church Times*, but I havn't read it yet.

TUESDAY, FEBRUARY 1ST.—Really this a *horrid* place! They wanted to make me get up at half-past six, but, of course, I hadn't had *half* enough sleep, so I wouldn't. One *can't* do good work if one hasn't had enough rest.

LATER.—I have seen the matron, who says I had better leave. I don't think this a nice hospital at all, so I told her I would, and asked her to have a hansom called for me.

### Where to Go.

ASYLUM WORKERS' ASSOCIATION.—The annual general meeting will be held on Monday, March 28th, at four p.m., in the rooms of the Medical Society, 11, Chandos Street. Sir James Crichton Browne will preside.

### What is Massage?

By a Hospital Sister.

This question continually meets the eye. The various answers intended to throw light on the subject are so vague, and so contradictory, that it is time that some definite reply should be given as to what the term massage really means. It is not a haphazard and unscientific "rubbing," nor is it the shampooing which obtains at the Turkish baths, though to the general public the difference may seem very slight.

It is an art, and it is by no means easy to attain skill as an operator; on the contrary, it requires long training and long practice, and a masseuse should have a more serious course of study than is usually the case in this country.

The object of this paper is, therefore, not to teach massage —which can only be learned by experience—but to explain its difficulties, and to show its usefulness.

Massage consists of a series of delicate and intricate manipulations; each of them is performed with a definite object that must be clearly borne in mind by the operator.

1. The first movement (Effleurage) is done with the specific intention of improving the circulation. It consists of long sweeping strokes, necessarily with and not contrary to the flow of the blood. This prepares the member for the stimulating movement, termed friction, and is also resorted to between each different manipulation of the series.

2. Friction is a firm yet delicate circular movement, and is one which the patient finds particularly agreeable. It generates heat and aids absorption, and must also be done centripetally.

3. Petrissage. This movement, certainly the most difficult, and perhaps the most effective of all, is a kneading of individual muscles, and is especially performed on those surrounding any affected part. In the case of an injured joint it is applied with the thumbs, not only to the muscles, but to the capsules and such ligaments as can be reached. This gives tone to the muscles, and helps to disperse waste material.

4. Tapotement is a sharp stinging movement, stimulating the nerves, and acting beneficially on the capillary circulation. There are various forms of tapotement, but it is not my intention to enter into details.

From this it will be seen that massage practically amounts to passive movement, and is therefore invaluable for those unable to take other exercise.

Having explained as briefly and as simply as possible the theory of massage, it is obvious that to carry out that theory proper y systematic training is required, and it is much to be deplored that so little time is devoted to the purpose in this country. More study ought to be given to elementary anatomy and more time to practical work. There is a vast difference between knowing what the "movements" are and being a skilful operator. It will take a year of steady practice to make the left hand as useful as the right, so that a patient can feel no difference in the touch or grasp of either hand; to make the hands and fingers thoroughly supple, and to give the instinctive feeling of how much or how little pressure should be used. One of the difficulties that pupil masseuses have to contend with, viz., learning how to treat stiff joints, curvature of the spine, &c., on a normal subject, ought to be met by arrangement with the various hospitals, the pupil masseuses when sufficiently advanced giving their services in return for the great boon the practice would be, before taking up private practice.

With regard to the training abroad, by far the most thorough is to be obtained at the Central Institute of Gymnastics in Stockholm. As may be gathered from the name of this institution, massage is only one branch of the great system of physical education taught there. Sweden is undoubtedly the home of physical culture, by which we mean not the pretty tricks and graces so often taught under that name, but the full proportionate development of the powers of the human body. As this is only to be obtained by systematic exercises, it is conceivable that when some part of the body has been hindered in its development by disease a carefully-planned scheme of exercises should restore it to its normal condition.

The exercises employed to cure, or at least to alleviate, various ailments (the so-called medical or curative gymnastics) are divided into active and passive movements; under the latter heading we find massage.

Every student at the institute is obliged to attend the whole course, and no one is allowed to take up one subject (such as massage) by itself. The curriculum includes anatomy, physiology, elementary pathology, medical and school gymnastics (theoretical and practical).

Both men and women are trained at the institute; the course for the former is of three years' duration, as military gymnastics (fencing, &c.) are included in their teaching. The women's lasts for two years only.

Twenty-five is the maximum number of women admitted to each course, twenty Swedes and five foreigners. They must produce good certificates of health and education, and be between the ages of twenty-five and thirty-five. The competition for admission is now so keen that most girls wishing to enter the institute qualify themselves by taking an examination which corresponds with our matriculation.

The routine of practical work is as follows: Each student takes daily four or five patients in the clinical department, develops her own physique by an hour's gymnastic exercise every morning, and also in teaching a class at one of the girls' schools in the neighbourhood.

In every Swedish school gymnastics are compulsory unless a doctor exempts a child from the ordinary class. In most schools the children's backs are examined every term, and if there be the slightest inclination to spinal curvature or other defect they are ordered a course of curative exercises until they are in a condition to join their comrades again. Many of the poorer children are treated free of charge at the institute, and a number of poor women and children suffering from various ailments are sent in by doctors from all parts of Sweden.

The clinical department is under the supervision of a medical man, who also instructs the students in anatomy and kindred subjects.

There are other points of interest in the Swedish system of training, but space does not admit of a more detailed account.

(*To be continued.*)

### Minor Appointments.

Eastern Hospital, Homerton. — The following have been appointed Charge Nurses at the above institution: Miss Birdsall Hopkinson, who was trained at the Brownlow Hill Infirmary, Liverpool, and has since worked under the Metropolitan Nursing Association, and as nurse matron of Fitbury Cottage Hospital; Miss A. Cummins, who was trained at the Mater Misericordæ Hospital, Dublin; Miss M. E. Cruice, who was trained at the same hospital as Miss Cummins; and Miss M. J. Burrell, who was trained at the Radcliffe Infirmary, Oxford, and has since held the post of head nurse at the District Asylum, Monaghan.

St. Pancras Infirmary, Dartmouth Park Hill, N.— Miss Mawby, who was trained at St. Marylebone Infirmary, has been appointed Charge Nurse of this infirmary.

### The Book World for Women and Nurses.

[We invite Correspondence, Criticism, Enquiries, and Notes on Books likely to interest Women and Nurses. Address, Editor, THE HOSPITAL (Nurses' Book World), 28 & 29, Southampton Street, Strand, London, W.C.]

DISTRICT NURSING ON A PROVIDENT BASIS. By JAMIESON B. HURRY, M.A., M.D. (The Scientific Press, Limited, 28 and 29, Southampton Street, Strand, London, W.C. 1898; pp. 98. Price 2s.)

This useful little work and the system it advocates are deserving the attention of all thoughtful people to whom the problem of relieving sickness in the most efficient way is a constantly recurring one. As the author justly remarks (page 18), "There is no more reason why skilled service should be given in the form of free nursing than in the form of free medical attendance, and especially when organised on a large scale and applied indiscriminately; the effect is to pauperise and to injure the moral fibre of the people." There are few persons, we venture to think, so utterly destitute as not to be able to afford a small yearly contribution towards providing that help which has become such a necessity in all cases of illness nowadays. Of course, there are cases where this is impossible, and for these there is the workhouse infirmary. The question of the expediency of relieving the destitute sick in their own homes is a vexed one, and not likely to be very readily entertained by the opponents of outdoor relief, of which it is a variety. The principle, therefore, which encourages thrift and forethought among the poorer population is to be welcomed and encouraged. At Heathfield the payment by labourers of 1s. yearly and 4s. for maternity nursing cannot be described as excessive, while 2d. to 6d. a visit for non-members is within the reach of the majority of those whom the nurses' services are destined to benefit. Payments must necessarily vary in different districts, but that some small amount should be contributed by the poor themselves as well as by the rich in their behalf is most desirable. We should like to have heard some arguments advanced in favour of paying district nurses more liberal salaries. The present basis of £100 per annum is very little, when out of it the nurse is expected to find her own board, lodging, uniform, and washing. Until larger salaries are offered, there will always be a difficulty in inducing the more highly qualified women to come forward for this branch of work, and surely nowhere are they more sorely needed than in the cottages of a scattered population where emergencies arise and have to be promptly dealt with until such time as medical aid arrives. In conclusion, we have every confidence in recommending this little work to those who are interested in the subject of district nursing. Its reasoning is clear and the resulting deductions sound and convincing. We wish it were possible to place all district nursing on a provident basis, however small, as a matter of principle, apart altogether from expediency.

THE RETURN TO THE CROSS. By the Rev. W. ROBERTSON NICOLL, M.A., LL.D., Editor of "The Expositor," "The Expositor's Bible," &c. (London: Isbister and Co., Limited, 15 and 16, Tavistock Street, Covent Garden. 1897.)

Those who know Dr. Robertson Nicoll merely as the discoverer of the "Kailyard" will hardly expect to find in him not merely a theologian, but one who holds most strongly to the need of a fixed and definite creed. This is a very beautiful and strengthening book of devotional addresses, based on the firmest yet most reasonable conviction of the truth and importance of the articles of the Christian faith. Dr. Nicoll condemns sternly those who regard Christianity as merely a synonym for an easy-going amiability, those who, as Liddon put it, instead of saying, "straight is the gate and narrow is the way, and few there be that find it," say "wide is the gate and broad is the way, and no one can reasonably miss it." In a review of Miss E. S. Phelps' book, "A Singular Life," the writer says, "As for those whose Gospel is that Christ lived once in Palestine, and that there are valuable sentences to be traced to Him, and that with a little care almost all the familiar verses of the New Testament can be used with the original meaning taken out of them, it needs no prophet to foretell the fate of a church controlled by such. It will live just as long as it is not found out. When it is found out it will be swept from the earth as an organised hypocrisy." In this review he speaks very strongly of the importance of accepting those doctrines which at present many people seem to think are in no way essential to Christianity. There is no valid basis of faith but theology, he says, in effect:—

"Who would preach the Gospel of the love of Abraham or of Moses or of Paul? What is there about Christ that puts Him in an altogether different category from theirs, and gives Him power to help us to-day? The answer is, we suppose, that Christ is God. Is not this theology? What happened after Christ's death that gave Him power to reach us with His love as the others cannot. He rose again. Is not this also theology? We seem to be dealing already with two articles of the Christian creed, the Incarnation and the Resurrection, for without these the proclamation that Christ loves the human souls that live is a mockery. And how do we know Christ loves us? The answer, we suppose, is that His heart was revealed in the Cross, and does not that give us the doctrine of the Atonement? . . . Nor is that the whole. It may be answered, We know that Christ loved us by the gracious words that proceeded out of his mouth; was it not He who said, 'Let not your heart be troubled, neither let it be afraid'? But then did He say these words, or were they the invention of some other? Is not this that very question of the authenticity of the fourth Gospel of which Miss Phelps speaks with such magnificent scorn?"

Elsewhere Dr. Nicoll defends the unpopular doctrine of justification by faith, and the conception of prayer as being a real request which expects and will receive a real response, and argues out the reasonableness of these parts of the creed in an earnest and convincing way. "The Return to the Cross" should be valuable to many who find no help in the idea of a merely human Christ, yet fail to see any reasonable explanation of His divinity.

### Everybody's Opinion.

[Correspondence on all subjects is invited, but we cannot in any way be responsible for the opinions expressed by our correspondents. No communication can be entertained if the name and address of the correspondent is not given, as a guarantee of good faith but not necessarily for publication, or unless one side of the paper only is written on.]

### NURSES' AMBITIONS.

"I SPY" writes: I should much like to ask what are considered honourable ambitions for nurses. Of course, most of us would like to arrive at the dignity of matron, or, failing that, to make a nice little competence in private nursing, or to own a nursing home; and we know many who drop out of the running by not knowing exactly what they would like to become. It seems to me that if we nurses were to consider fairly what we wanted to do in the early days of our training there would be fewer failures, and if nurses who have succeeded or failed in reaching the goal they set out for would give some of their experiences in your columns it would be helpful to the younger ones who are just starting on the way. Perhaps there are some who will not be altogether unwilling to let others learn from their experience, and I, for one, should much like to hear what ambitions are considered honourable, and how it is that some nurses succeed and others fail.

### Notes and Queries.

**The contents of the Editor's Letter-box have now reached such unwieldy proportions that it has become necessary to establish a hard and fast rule regarding Answers to Correspondents. In future, all questions requiring replies will continue to be answered in this column without any fee. If an answer is required by letter, a fee of half-a-crown must be enclosed with the note containing the enquiry. We are always pleased to help our numerous correspondents to the fullest extent, and we can trust them to sympathise in the overwhelming amount of writing which makes the new rules a necessity. Every communication must be accompanied by the writer's name and address, otherwise it will receive no attention.**

*Training in a Homœopathic Hospital.*

(200) Is a three years' training and certificate from a homœopathic hospital as valuable in the nursing profession as that from any other hospital?

Probably. Nurses are not trained in medicine, but in nursing.

*Brompton Consumption Hospital.*

(201) How can I get a consumptive man into Brompton Consumption Hospital?—*Nurse Burrell.*

You must get a letter from a subscriber. Perhaps the secretary would tell you how best to obtain it.

*Private Nursing on the Riviera.*

(202) Please tell me of a home on the Riviera where an English nurse could obtain private work and take her own fees? What place affords the best opening for maternity work?—*Nurse M. E. F.*

Co-operative nursing homes are not to be found in the Riviera. Miss Bryant has a nursing institute at 19, Via Vittorio Emanuele San Remo; and Mrs. Dan Norris, Villa D'Ormesson, Cannes, occasionally requires nurses for her invalid home. A connexion is everywhere difficult to work up, and more so on the Continent than in England. Be careful to make sure of a living before embarking on such a venture.

*Ambulance Lectures.*

(203) Are there any ambulance lectures being given in London just now, and what is the fee?—*H. R.*

Write to the Secretary, St. John Ambulance Association, St. John's Gate, Clerkenwell, E.C. All queries requiring a private reply must be accompanied by a fee of 2s. 6d.

*Mental Nursing.*

(204) Could you give me any information as to where I would be trained for mental nursing? England preferred.—*A Constant Reader of* The Hospital.

In about 80 different asylums. The County Asylum, Lancaster, might suit you. The Berrywood Asylum, Northampton, grants certificates and is noted for training. The County Asylum, Dorchester, also offers good training and a certificate.

*Syncope.*

(205) Please tell me if there is a work specially devoted to syncope? We are nearly two miles from a doctor, and the patient is old.—*Ann H.*

There is no book specially devoted to syncope. The general rules may be learnt from any good nursing book, such as that by Lewis (3s. 6d.), the Scientific Press, 28 & 29, Southampton Street, Strand, London, W.C. Ask the doctor next time you see him if the case calls for any special treatment. You should have sent full name.

*Earcaps.*

(206) Please tell me where I can get earcaps for prominent ears?—*Nancy.*

Earcaps may be obtained from the makers, Messrs. C. Claxton, 62, Strand.

*Fifty Beds.*

(207) Will you kindly inform me if a hospital containing 50 beds is a recognised training school for probationers, and if, after two years' training in such, the nurse would be eligible for head nurse in other hospitals or under the Local Government Board?—*Matron.*

Section 3 Article 3 of the L.G B. runs: "Any superintendent nurse . . . shall . . . be a person qualified for the appointment by having undergone for *three* years at least a course of instruction in the medical and surgical wards of any hospital or infirmary being a training school for nurses, and maintaining a *resident physician* or *house surgeon.*" The italics are ours. If "Matron's" hospital has a resident medical officer, either physician or surgeon, and she chooses to train probationers for three years, the certificate will be recognised by the L.G.B. "Matron" ought to have sent her name.

*Homes for Poor Ladies.*

(208) Would you kindly inform me if there is a home where a poor lady could be received for £40 a year?—*K. J.*

Apply the Trustees Ballard's Home for Widows, 43, Newton Road, Westbourne Grove, London; the Hon. Secretary of Belmont Home for Gentlewomen, Lewisham Park, 6, Walerand Road, Lewisham Hill, S.E.; the Secretary Church Association, 14, Buckingham Street, Strand, W.C., for particulars of Luther Memorial Home, 120, Ledbury Road, Bayswater. There are several others. "K. J." should have given fuller particulars.

*Civilian Hospital at Alexandria and Cairo.*

(209) Will you kindly tell me if there is an English civilian hospital at Alexandria or at Cairo? If so, have they English matrons and nurses?

There is no exclusively British hospital in Egypt. At Cairo there is the Kasr-el-aini Hospital, with Miss Angles for head nurse and six English sisters, and the Deaconess Victoria Hospital, under a committee of eight, composed of two Englishmen, two French, two Swiss, and two from the American colonies. The matron is Sister Superior Elise Brakabusch.

*Cast-off Uniforms.*

(210) Could you tell me where I could buy nurses' left off uniforms for charitable purposes?—*A. Y.*

We are not aware of any agency for this purpose.

*Nursing in France.*

(211) Can you give me any information respecting hospital or private nursing in France? I am a certificated nurse, with good testimonials, and want to nurse in France. but not in an English hospital or institute.

Are you reading the articles entitled "Nursing in Paris Hospitals," which began in the Mirror for February 12th? We learn on excellent authority that the conditions of nursing in French institutions would be insupportable to English women, and that the market for private work is overcrowded.

*Children's Nurse.*

(212) Will you kindly tell me if there is another institute for training lady nurses for children beside the Norland Home?—*Anxious One.*

That, we believe, is the only one in London.

*Peptonise Milk.*

(213) How is milk peptonised? I have tried carefully, but mine curdles when boiled.—*Puzzled.*

The following recipe from a nurses' manual may be useful. Boil a pint of *fresh* milk with a quarter pint water, and allow it to cool to 140 *deg. F.*, add the contents of a zymine peptonising tube (Fairchild's), mix, and allow to stand in a warm place for *ten* minutes, boil. The italics are ours, as "Puzzled's" milk curdled probably from the neglect of one or other of the points thus emphasised.

*A Recovered Mental Patient.*

(214) Can you tell me how to obtain work for a recovered mental patient. She is a refined woman, a trained nurse, and strong. Is there not an association for helping such cases?—*Scot.*

Can any of our readers help "Scot" in this? Work for such patients is most difficult to obtain, as the After-Care Association, Church House, Westminster, finds and reports.

*Books and Training.*

(215) Will you please recommend to me the best and most complete book on nursing? What steps should I take in order to enter one of the large London hospitals (St. Bartholomew's, Guy's, or St. Thomas's) as probationer?—*M. T.*

1, Comparisons are odious! "Nursing," by Isabel Hampton (7s. 6d.), is widely used, so is "Nursing, its Theory and Practice," by Lewis (3s. 6d.), both to be obtained of the Scientific Press, 28 & 29, Southampton Street, Strand. "How to Become a Nurse," by H. Morten (2s. 6d.), same publishers, will answer your second question.

*How to Start a District Nurse.*

(216) Will you kindly tell me how to start a district nurse for the country? The idea is to have two nurses, and take a cottage for them, for a wide country district. If you will insert an answer in the next edition of The Hospital I shall be much obliged. Also kindly state the address of the Jubilee Nurses' Institution.—*E. A. B.*

The address of the Queen Victoria Jubilee Institute for Nurses is 1, St. Katherine's Hospital, Regent's Park, N.W. Perhaps some of our correspondents would give their experience on this subject.

*S. A. F.* had better consult a hospital surgeon of experience in the treatment of deformities. The "silver bridge" referred to is a sort of splint used in the treatment of such cases, but is not in itself a "treatment."

### Appointments.

### MATRONS.

Victoria Hospital for Children, Hull.—Miss J. Watson was appointed Matron of this hospital on March 3rd. She was trained at the Infirmary, Leeds, and was for two years night superintendent at Charing Cross Hospital, and for three years home sister and assistant matron at the Victoria Hospital for Children, Chelsea.

North-Western Fever Hospital, Haverstock Hill.—Miss Louisa Basan, who was trained at St. Bartholomew's Hospital, has been appointed Assistant Matron of the above. She has been charge nurse and afterwards night superintendent at Fountain Fever Hospital, Tooting.

Cottage Hospital, Petersfield.—Miss Julia Redding was appointed Matron of this hospital on March 14th. She was trained at Guy's Hospital, and afterwards joined the Wigmore Institution for private nursing, where she has been for several years.

Oriolet Hospital, Loughton, Essex.—Miss Clare Crowther has been appointed Matron of this hospital. She was trained at the Homœopathic Hospital, Bloomsbury.

